# High-Fat Diet-Induced Insulin Resistance Does Not Increase Plasma Anandamide Levels or Potentiate Anandamide Insulinotropic Effect in Isolated Canine Islets

**DOI:** 10.1371/journal.pone.0123558

**Published:** 2015-04-09

**Authors:** Orison O. Woolcott, Joyce M. Richey, Morvarid Kabir, Robert H. Chow, Malini S. Iyer, Erlinda L. Kirkman, Darko Stefanovski, Maya Lottati, Stella P. Kim, L. Nicole Harrison, Viorica Ionut, Dan Zheng, Isabel R. Hsu, Karyn J. Catalano, Jenny D. Chiu, Heather Bradshaw, Qiang Wu, Richard N. Bergman

**Affiliations:** 1 Diabetes and Obesity Research Institute, Cedars-Sinai Medical Center, Los Angeles, California, United States of America; 2 Department of Physiology and Biophysics, Keck School of Medicine, University of Southern California, Los Angeles, California, United States of America; 3 Department of Animal Resources, University of Southern California, Los Angeles, California, United States of America; 4 Department of Psychological and Brain Sciences, Indiana University, Bloomington, Indiana, United States of America; CRCHUM-Montreal Diabetes Research Center, CANADA

## Abstract

**Background:**

Obesity has been associated with elevated plasma anandamide levels. In addition, anandamide has been shown to stimulate insulin secretion *in vitro*, suggesting that anandamide might be linked to hyperinsulinemia.

**Objective:**

To determine whether high-fat diet-induced insulin resistance increases anandamide levels and potentiates the insulinotropic effect of anandamide in isolated pancreatic islets.

**Design and Methods:**

Dogs were fed a high-fat diet (n = 9) for 22 weeks. Abdominal fat depot was quantified by MRI. Insulin sensitivity was assessed by the euglycemic-hyperinsulinemic clamp. Fasting plasma endocannabinoid levels were analyzed by liquid chromatography-mass spectrometry. All metabolic assessments were performed before and after fat diet regimen. At the end of the study, pancreatic islets were isolated prior to euthanasia to test the *in vitro* effect of anandamide on islet hormones. mRNA expression of cannabinoid receptors was determined in intact islets. The findings *in vitro* were compared with those from animals fed a control diet (n = 7).

**Results:**

Prolonged fat feeding increased abdominal fat content by 81.3±21.6% (mean±S.E.M, P<0.01). *In vivo* insulin sensitivity decreased by 31.3±12.1% (P<0.05), concomitant with a decrease in plasma 2-arachidonoyl glycerol (from 39.1±5.2 to 15.7±2.0 nmol/L) but not anandamide, oleoyl ethanolamide, linoleoyl ethanolamide, or palmitoyl ethanolamide. In control-diet animals (body weight: 28.8±1.0 kg), islets incubated with anandamide had a higher basal and glucose-stimulated insulin secretion as compared with no treatment. Islets from fat-fed animals (34.5±1.3 kg; P<0.05 versus control) did not exhibit further potentiation of anandamide-induced insulin secretion as compared with control-diet animals. Glucagon but not somatostatin secretion *in vitro* was also increased in response to anandamide, but there was no difference between groups (P = 0.705). No differences in gene expression of CB1R or CB2R between groups were found.

**Conclusions:**

In canines, high-fat diet-induced insulin resistance does not alter plasma anandamide levels or further potentiate the insulinotropic effect of anandamide *in vitro*.

## Introduction

The endocannabinoid system plays a fundamental role on the regulation of appetite and energy expenditure, and overall in the pathogenesis of obesity [1, 2]. Anandamide and 2-arachidonoyl glycerol (2-AG) are two of the major endogenous ligands of cannabinoid receptors 1 (CB1R) and 2 (CB2R) [3]. Besides their known central effects on appetite regulation, these ligands have been shown to have peripheral metabolic effects on several tissues [1, 4]. For example, endocannabinoids induce biosynthesis of fatty acids and triglycerides in the liver. In the adipose tissue, endocannabinoids promote cell differentiation, inhibit lipolysis and stimulate glucose uptake [2, 5].

Typically, obesity is associated with hyperinsulinemia and insulin resistance [6]. As a compensatory mechanism, insulin secretion is enhanced in response to insulin resistance to prevent hyperglycemia [7, 8]. Failure of the β-cell to secrete adequate amounts of insulin to compensate for insulin resistance may contribute to the pathogenesis of type 2 diabetes [9]. Interestingly, plasma anandamide levels have been reported to be elevated in obese individuals [10, 11]. In addition, pancreatic anandamide content has been shown to be increased in diet-induced obese rodents compared with lean animals [12]. Moreover, *in vitro* studies have shown that anandamide stimulates basal insulin secretion in isolated pancreatic islets from lean rats [13] and potentiates glucose-stimulated insulin secretion (GSIS) in islets from non-obese humans and rats [13, 14]. Thus, it has been proposed that the endocannabinoid anandamide might contribute to hyperinsulinemia in response to fat diet or obesity [15, 16], to compensate for insulin resistance.

Independent studies in rodents and humans have shown a direct association between plasma anandamide levels and obesity [10, 11, 17]. However, no previous study has specifically determined the effect of high-fat diet-induced insulin resistance on plasma anandamide levels. Moreover, although *in vitro* studies has shown a positive effect of anandamide in insulin secretion [13, 14], whether diet-induced insulin resistance potentiates the insulinotropic effect of anandamide on the β-cells remains unknown. In the present study, we used a canine model to specifically test the hypothesis that high-fat diet-induced insulin resistance increases plasma anandamide levels and that insulin resistance further potentiates the insulinotropic effect of anandamide *in vitro*. Given the ethical concerns to test these hypotheses in humans, and the limitations to measure numerous biochemical parameters longitudinally in rodents, we used a well established canine model of high-fat diet-induced insulin resistance, a fully validated large animal model exhibiting marked insulin resistance and abdominal fat accumulation [18–21]. Our findings in canines indicate that high-fat diet-induced insulin resistance does not alter plasma anandamide levels or potentiate the insulinotropic effect of anandamide *in vitro*.

## Materials and Methods

### Animals

The present study was conducted in adult male mongrel dogs, 1–3 years old. We included a subset of animals herein from a previous study [19] to report more extensive data related to β-cell function *in vivo* and plasma levels of endocannabinoids. Animals were housed in kennels at the vivarium of the Keck School of Medicine, University of Southern California (Los Angeles, CA). The protocol for this study was submitted to, and approved by, the ethics committee of the University of Southern California, and all procedures followed the regulations from the Institutional Animal Care and Use Committee.

### Diet

At arrival, animals started a standard diet consisting of 825 g of dry chow (a mixture of Laboratory High Density Canine Diet and Prolab Canine 2000, Richmond, IN) for 2–3 weeks. Diet was switched to a weight-maintaining control diet for 3 weeks consisting of 825 g of dry chow and one canned food (Hill’s Pet Nutrition, Topeka, KS). Total daily food presented contained 3,582 kcal (28.1% from proteins, 31.3% from fat, 40.6% from carbohydrates). After body weight stabilization, animals were fed a hypercaloric high-fat diet (HFD) for 22 weeks. HFD consisted of a control diet enriched with lard/bacon grease (6 g/kg of baseline body weight). Total daily calorie content of HFD presented consisted of 5,527 kcal (53.0% from fat). This HFD regimen has been extensively validated in our laboratory to effectively and reproducibly induce insulin resistance in canines [18–21]. Food was presented from 09:00–12:00 h. Water was provided *ad libitum*. Since experiments *in vitro* were conducted at the end of the study, our findings *in vitro* were compared with those from animals fed a control diet for 4–6 weeks (n = 7). The calorie content of the control diet was estimated based on the personal experience of our veterinary staff on mongrel dogs.

### Assessment of metabolic parameters

A group of 9 animals were fed a HFD. Body weight, abdominal fat content, β-cell function *in vivo*, whole-body insulin sensitivity, and biochemical analyses (fasting glucose, insulin, C-peptide, glucagon, plasma non-esterified fatty acids, anandamide, 2-AG, oleoyl ethanolamide, linoleoyl ethanolamide, and palmitoyl ethanolamide), were determined before and after HFD. Further metabolic assessments have been published elsewhere [19, 20].

### Abdominal fat composition

Fat accumulation in the abdominal region was quantified by MRI using a 1.5-T Gemsow Scanner (General Electric). MRI scan included eleven 1-cm thick horizontal slices. Fat area (visceral and subcutaneous adipose tissue) of each slice was estimated based on pixel intensity [19]. Abdominal fat volume was calculated from the product of the fat area and the thickness of the slice. Total abdominal fat volume included the sum of the fat volume obtained from the 11 slices.

### Insulin sensitivity ***in vivo***

Whole-body insulin sensitivity was estimated by the euglycemic hyperinsulinemic clamp, as previously described [20]. Basal samples were taken at t = -30, -20, -10, and -1 min. At t = 0 min, a somatostatin infusion was started and continued for the duration of the experiment. Porcine insulin was infused into a peripheral vein to induce hyperinsulinemia. Glucose was clamped at basal concentration by a variable infusion of glucose. Blood samples were collected every 10 min from t = -30 to 60 min, every 15 min from t = 60 to 120 min, and then every 10 min from t = 120 to 180 min. Whole-body insulin sensitivity (SI_CLAMP_) was calculated from the following equation: SI_CLAMP_ = ΔGINF/(ΔI X G), where ΔGINF is the difference in glucose infusion rate during the steady state period (t = 150–180 min) from basal, ΔI is the difference in plasma insulin at steady state from basal, and G is the steady-state plasma glucose concentration.

### β-Cell function ***in vivo***

Assessment of *β*-cell function *in vivo* was performed using the graded-hyperglycemic clamp [18, 22]. Glucose infusion (50%) was injected peripherally at variable rates using a syringe pump (Razel Scientific Instruments, Stamford, CT) in order to maintain blood glucose constantly at three sequential concentrations: 5.6 (*t* = 0–59 min), 8.3 (*t* = 60–149 min) and 11.1 mmol/L (*t* = 150–240 min). Blood samples were collected every 10 min throughout the experiment, starting 20 min prior to the commencement of glucose infusion. Glucose infusion rates were adjusted periodically based on plasma glucose readings using a blood glucose analyzer (YSI 2700, Yellow Springs Instruments, Yellow Springs, OH). β-Cell function was measured as the slope of the relation between insulin (pmol/L) and glucose (mmol/L) during the steady-state at each glucose clamp period (*t* = 40−60 min: 5.6 mmol/L, *t* = 130−150 min: 8.3 mmol/L, and *t* = 210−240 min: 11.1 mmol/L).

### Islet experiments

Islets were obtained from control-diet and HFD dogs (after completing their corresponding diet periods, as described before), immediately before euthanasia, under general inhalant anesthesia. All animals were 2–3 years old at the time of the pancreas procurement. Islet isolation, islet viability stain, and assessment of β-cell function *in vitro* were performed as previously described [23].

### Static incubation

After 18–24 h culture, islets sized ~150–200 μm were handpicked and put on 24-well culture plates. Static incubation experiments consisted of 1-h equilibrium period with 3 mmol/L glucose, followed by 1-h additional period with either 3 mmol/L glucose (basal) or 15 mmol/L glucose (GSIS), in the presence or absence of exogenous anandamide and/or cannabinoid receptor antagonists. β-Cell function *in vitro* was estimated as the rate of insulin secreted (pmol•L^−1^/islet/h) during the second hour of static incubation (basal insulin concentration during the equilibrium period was subtracted for calculation) and also expressed as the stimulation index: ratio of the insulin secreted at 15 mmol/L glucose (second hour of static incubation) to the basal insulin secreted at 3 mmol/L glucose (equilibrium period) [23].

To explore possible paracrine effects of anandamide *in vitro*, insulin, glucagon, and somatostatin secretion were measured in same islets during short incubation period (1 h, 100 islets per well) and prolonged static incubation (14 h, 15 islets per well). Since basal hormone secretion did not yield detectable hormone concentrations of glucagon and somatostatin, for this purpose, secretion of islet hormones were expressed as the total amount secreted during the 1-h equilibrium period and the second period (either 1 h or 14 h). Insulin secretion was expressed as pmol•L^−1^/islet; glucagon and somatostatin release were expressed as ng•L^−1^/islet.

### Islet perifusion

Islet perifusion experiments were performed as previously described [23], with little modification. Islets were plated onto coverslips coated with BD cell-tak (BD Biosciences, San Jose, CA). After a 60-min equilibrium period (t = 0), islets were stimulated with 15 mmol/L glucose and 10 nmol/L anandamide for 36 min. The effluent was collected at 1-min intervals from t = −3 min until t = 10 min, followed by 2-min sampling until the end of the experiment (t = 36 min). β-Cell function was expressed as the net rate of insulin secreted (pmol•L^−1^/islet) in response to 15 mmol/L glucose relative to the lowest insulin concentration in the first phase (t = 0 to 8 min). Also, fold increase over average baseline was calculated. Second phase was defined as the period t = 9 to 36 min.

### Chemicals

Anandamide was purchased from Sigma-Aldrich (St Louis, MO). Iodoresiniferatoxin and AM630 were purchased from Tocris Bioscience (Minneapolis, MN). Rimonabant was kindly provided by Sanofi-aventis. All cannabinoid drugs were dissolved in dimethyl sulfoxide (0.1% final concentration).

### Biochemical assays

Plasma non-esterified fatty acids were measured using a colorimetric assay [18]. Insulin from plasma samples and *in vitro* experiments were determined by ELISA [23]. Plasma C-peptide (canine kit, Millipore, St. Charles, MO) was determined by radioimmunoassay in duplicate. *In vitro* glucagon (canine kit, Millipore) and somatostatin were determined by radioimmunoassay in single samples. Initial experiments were assessed using the somatostatin kit 13-RB306 (American Laboratory Products Company, Windham, NH), then switched to RK-060-14 (Phoenix Pharmaceuticals Inc., Burlingame, CA) because kit 13-RB306 was discontinued. Anandamide, 2-AG, oleoyl ethanolamide, linoleoyl ethanolamide, and palmitoyl ethanolamide concentrations were determined in plasma samples using methanol and acetonitrile for extraction, and liquid chromatography/tandem mass spectrometry (LC/MS/MS system) for analysis, as described in detail elsewhere [24].

### RNA extraction and quantification of mRNA by real-time PCR

Gene expression of CB1R, CB2R, and the transient receptor potential vanilloid 1 (TRPV1) was assessed in intact fresh canine islets. Approximately 200 islets per animal were suspended in TRI Reagent and stored at ‒80°C. RNA was extracted from frozen islets using the Tri-Reagent Kit (Molecular Research Center Inc., Cincinnati, OH). Total RNA concentration was quantified by spectrophotometry, absorbance measured at 260 nm. The 260/280 nm absorption ratio of all preparations ranged between 1.8 and 2.0. RNA integrity was assessed by gel electrophoresis using agarose/ethidium bromide gel. First-strand cDNA was synthesized according to the manufacturer’s protocol, from 1 μg of total RNA using Superscript II (Invitrogen, Carlsbad, CA). Real-time PCR was performed on a Light-Cycler 2.0 instrument (Roche Applied science, Indianapolis, IN). The cDNA was amplified using ‘Universal probe system’ in a glass capillary in a final volume of 10 μL reaction mix containing 2.5 μL, 100-fold diluted cDNA, 2 μL LightCycler Taq-Man Master Mix buffer (Roche Applied Science, Indianapolis, IN), 1 μmol/L specific forward-reverse primers (CB1R forward: 5’-CCTGGTTCTGATCCTTGTGG-3’; CB1R reverse: 5’-ACCATAATCGCAAGCAGAGG-3’; CB2R forward: 5’-TACTTGCCCCTTATGGGATG-3’; CB2R reverse: 5’-ATCAGGGGGAAAAGCTCAG-3’; TRPV1 forward: 5’-GGGAACCAGGGAAAAGTTCT-3’; TRPV1 reverse: 5’-GAACTGTGAGGGCATCAAGC-3’) and 0.5 μL of universal probes (for CB1R, CB2R, and TRPV1 we used #3, #56, and #13, respectively). All primers and universal probes were designed in Roche Applied Science website (http://www.roche-applied-science.com). Light cycler was programmed as follows: 1) Pre-incubation, 10 min at 95°C; 2) denaturation, 50 cycles, 10 s at 95°C; 3) annealing, 40 s at 60°C, followed by extension, 1 s at 72°C. After 45–55 cycles they were cooled at 42°C for 30 s. The quantification of 18S rRNA was used for sample normalization using SYBER Green I kit. PCR was performed according to the manufacturer’s protocol (Roche Applied Science). The specificity of amplification was determined by melting curve analysis.

### Statistical analyses

Data were not normally distributed, as determined by the Shapiro-Wilk's W test. Data were expressed as means±S.E.M., unless otherwise indicated. Spearman correlation was used to determine bivariate association between *in vivo* metabolic variables. Wilcoxon matched pairs test was used to evaluate differences within groups and the Mann-Whitney U test was used for comparison between groups. Friedman test was used to compare the effects of multiple drugs (groups) on β-cell function in islet batches from same animals. Friedman test was followed by Wilcoxon test if P < 0.05. A mathematical model approach (mixed-effects linear regression [25]) was used to determine the possible interaction between insulin, glucagon, and somatostatin secretion in response to anandamide during static incubation, while accounting for the number of replicates, glucose concentration, and type of diet. Differences were statistically significant if P<0.05. All analyses were performed using Statistica (StatSoft Inc., Tulsa, OK, USA) and Stata/SE 10.0 for Windows (StataCorp LP, College Station, TX).

## Results

### High-fat diet increases body weight and abdominal fat depot without affecting fasting plasma endocannabinoids levels

**Fig 1:**
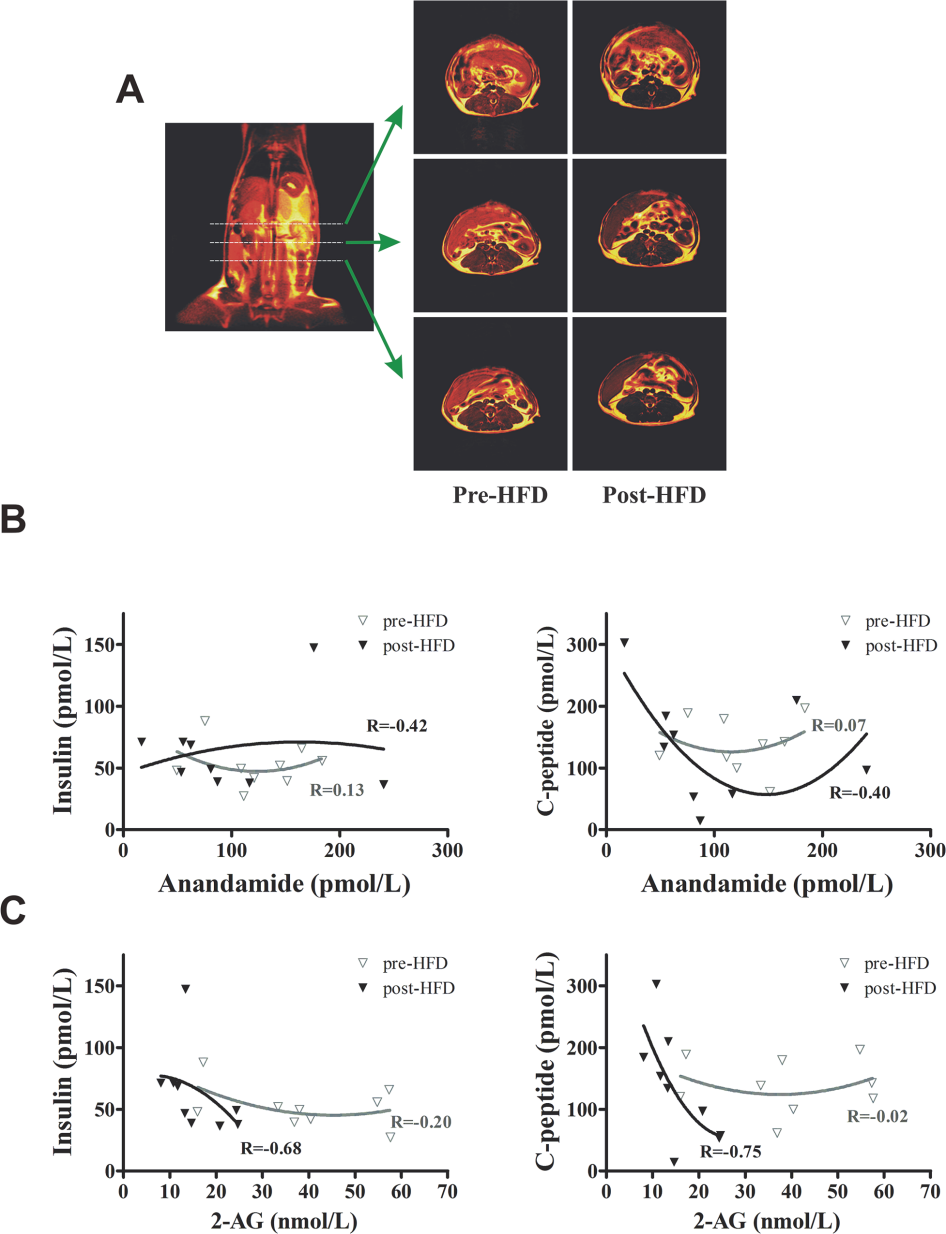
Metabolic changes in dogs maintained on a hypercaloric high-fat diet for 22 weeks. (**A**) Magnetic resonance scanning shows a substantial increase of fat content in the abdominal region after prolonged fat feeding. (**B**) Relationship between fasting plasma anandamide and insulin or C-peptide (**C**) Relationship between fasting plasma 2-AG and insulin or C-peptide. Association was determined using Spearman correlation. Lines represent non-linear (second-order polynomial) fit of the plots.

HFD for 22 weeks induced a significant increase in body weight by 13.4±2.5% (P<0.01), and total abdominal fat content by 81.3±21.6% (P<0.01) (Table 1 and Fig 1A). Likewise, HFD induced a marked reduction in insulin sensitivity by 31.3±12.1% (P<0.05). In addition, there was a decrease in fasting plasma 2-arachidonoyl glycerol but not anandamide, oleoyl ethanolamide, linoleoyl ethanolamide, or palmitoyl ethanolamide (Table 1). Overall fasting insulin and C-peptide did not significantly increase after prolonged fat diet given the variable response in the animals studied. However, additional analysis in a subset of animals showing consistent increase in fasting insulin (n = 5, from 55.0±10.4 to 78.9±18.3 pmol/L, P<0.05) revealed no increase in plasma anandamide levels (P = 0.893). Likewise, animals with increased fasting C-peptide (n = 5, from 138.0±14.8 to 196.7±29.5 pmol/L, P<0.05) showed no changes in anandamide (P = 0.50). Moreover, in animals with increased insulin slope during hyperglycemic clamp (n = 5, from 75.0±19.8 to 110.3±19.7 pmol•L^-1^/mmol•L^-1^, P<0.05), anandamide showed rather a tendency to decrease (from 122.8±22.5 to 78.6±11.6 pmol/L, P = 0.080).

**Table 1:** Profile changes in dogs (n = 9) maintained on a hypercaloric high-fat diet for 22 weeks.

	Week 0	Week 22	P
Body weight (kg)*	29.8 ± 1.2	33.9 ± 1.9	<0.01
Total abdominal fat depot (cm^3^)*	641.2 ± 85.3	1121.8 ± 194.7	<0.01
Glucose (mmol/L)*	5.39 ± 0.11	5.21 ± 0.07	0.173
Insulin (pmol/L)*	52.0 ± 5.8	63.0 ± 11.6	0.260
Insulin slope (pmol•L^-1^/mmol•L^-1^)	159.4 ± 43.4	123.8 ± 14.8	0.678
C-peptide (pmol/L)*	138.4 ± 14.8	133.9 ± 30.1	0.859
Glucagon (pmol/L)*	12.5 ± 1.0	9.0 ± 1.1	0.066
Insulin sensitivity (mg•kg^-1^•min^-1^)	6.5 ± 0.8	4.0 ± 0.5	<0.05
NEFA (mmol/L)*	0.85 ± 0.11	0.78 ± 0.10	0.767
Anandamide (pmol/L)*	123.3 ± 14.3	98.7 ± 23.3	0.441
2-AG (nmol/L)*	39.1 ± 5.2	15.7 ± 2.0	<0.01
PEA (nmol/L)*	6.9 ± 0.4	6.2 ± 0.6	0.086
LEA (pmol/L)*	301.0 ± 28.6	338.2 ± 76.9	0.767
OEA (nmol/L)*	13.3 ± 1.2	16.0 ± 2.9	0.374

Values are means±S.E.M. 2-AG, 2-arachidonoyl-glycerol; C-peptide slope, changes in plasma C-peptide relative to plasma glucose during hyperglycemic clamp; Insulin slope, changes in plasma insulin relative to plasma glucose during hyperglycemic clamp; LEA, linoleoyl-ethanolamide; NEFA, non-esterified fatty acids; OEA, oleoyl-ethanolamide; PEA, palmitoyl-ethanolamide; SI, whole-body insulin sensitivity assessed by the euglycemic hyperinsulinemic clamp.

* Fasting plasma values.

This study also found no correlation between body weight or abdominal fat adiposity and plasma anandamide or 2-AG. Likewise, we found no correlation between plasma anandamide and insulin or between plasma anandamide and C-peptide (Fig 1B). However, we found an inverse correlation between plasma 2-AG and insulin (P<0.05) and between plasma 2-AG and C-peptide (P<0.05) after 22 weeks of HFD (Fig 1C). Collectively, these findings do not support a direct association between fasting plasma anandamide and β-cell function *in vivo*.

### Supraphysiologic anandamide concentrations enhance insulin secretion ***in vitro***

**Fig 2:**
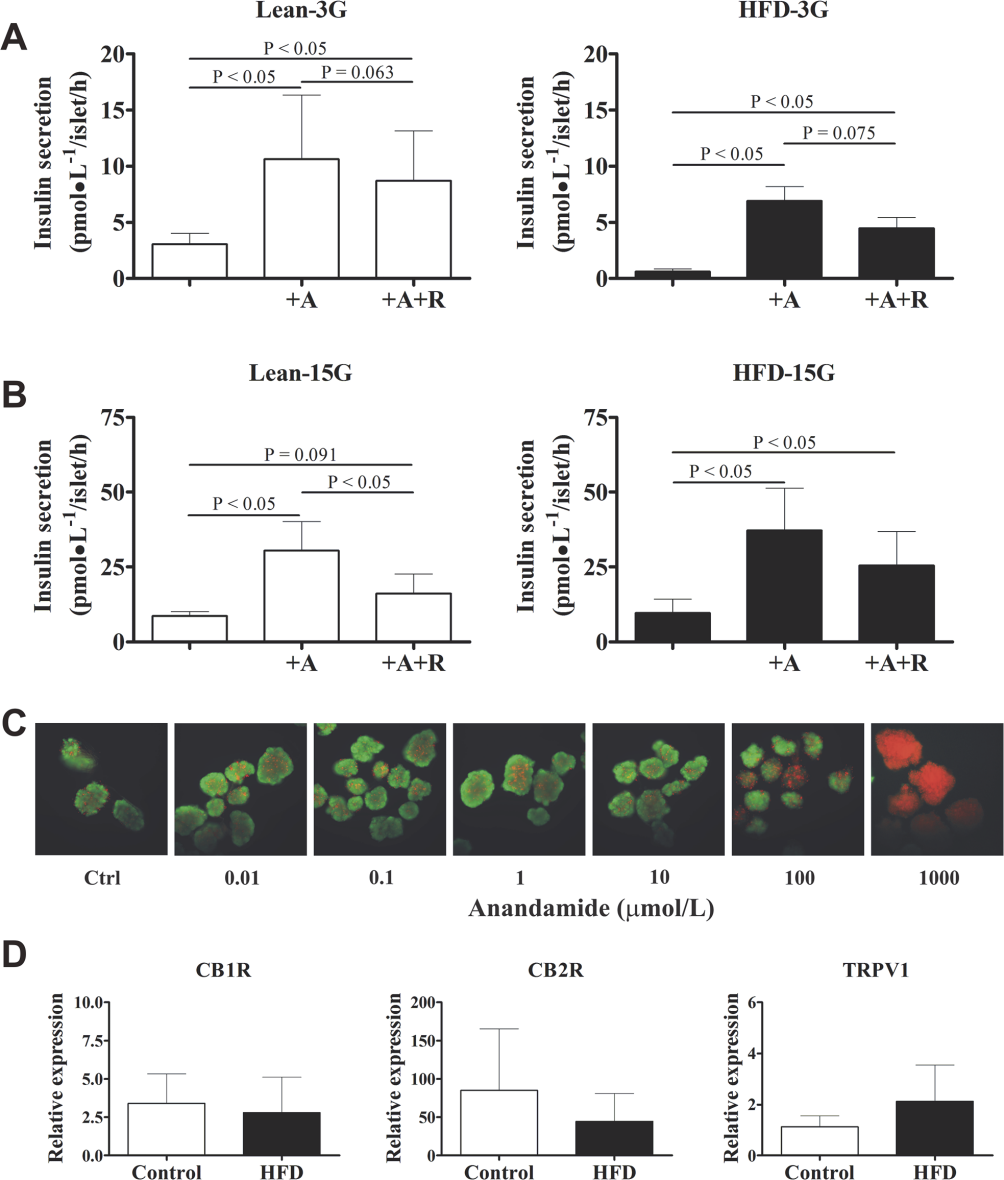
Supraphysiologic concentrations of anandamide enhance *in vitro* insulin secretion. (**A**) Anandamide stimulates basal insulin secretion in control-diet (n = 7) and high-fat diet (HFD) animals (n = 6) at 3 mmol/L glucose (3G). (**B**) Anandamide also potentiates glucose-stimulated insulin secretion in both groups at 15 mmol/L glucose (15G). Islets were incubated with anandamide or CB1R antagonist rimonabant (R) at 10 μmol/L for 1 h. Experiments on every animal were done in quadruplicate. Data are mean±S.E.M. (**C**) At the doses tested to stimulate insulin secretion, anandamide did not impair islet viability. Green and red colors represent viable and non-viable cells, respectively. Staining of islet batches from a same animal is representative of 3 independent experiments. Note the complete loss of islet viability (red stain) at 1000 μmol/L, consistent with a massive release of insulin as measured by ELISA (data not shown). Total magnification: 100X. (**D**) Relative mRNA expression of CB1R, CB2R, and TRPV1 to 18S in intact islets from control (n = 4) and HFD animals (n = 6–7).

Islets were isolated from control-diet dogs (n = 7, body weight: 28.8±1.0 kg) and from dogs fed a HFD for 19–22 weeks (21.5±0.5 weeks) (n = 6, body weight: 34.5±1.3 kg). In control dogs, islets incubated with 10 μmol/L anandamide had a higher rate of basal insulin secretion (10.6±5.7 pmol•L^−1^/islet/h) as compared with no treatment (3.0±1.0 pmol•L^−1^/islet/h; P<0.05) (Fig 2A). In HFD dogs, islets incubated with 10 μmol/L anandamide also had a higher rate of basal insulin secretion (6.9±1.3 pmol•L^−1^/islet/h) as compared with no treatment (0.6±0.2 pmol•L^−1^/islet/h; P<0.05). Despite the difference in body weight between groups (P<0.05), the insulinotropic effect of anandamide was not different (P = 0.886).

Anandamide also potentiated GSIS in control-diet and HFD animals (Fig 2B), without compromising islet viability (Fig 2C). In control dogs, islets incubated with anandamide had higher GSIS at 15 mmol/L glucose (30.5±9.7 pmol•L^−1^/islet/h) as compared with no treatment (8.6±1.5 pmol•L^−1^/islet/h; P<0.05). Likewise, the stimulation index (fold increase of insulin secretion over basal) was higher with anandamide (16.5±2.6 versus 5.9±0.6; P<0.05). The latter finding was not dependent on basal insulin differences (P = 0.499). In HFD dogs, islets incubated with anandamide also had higher GSIS (37.2±14.1 pmol•L^−1^/islet/h) as compared with no treatment (9.6±4.7 pmol•L^−1^/islet/h; P<0.05). The stimulation index was also higher with anandamide (21.1±7.0 versus 5.9±1.5; P<0.05), independent of basal insulin differences (P = 0.345). The insulinotropic effect of anandamide at high glucose concentrations was not different between HFD and control-diet dogs, either when comparing insulin secretion rates (P = 0.775) or the stimulation indexes (P = 1.00). Moreover, we found lower insulin secretion at low glucose concentrations in islets from HFD dogs (P = 0.010) as compared with islets from lean dogs (Fig 2A). However, we found no differences in GSIS (P = 0.568) between groups (Fig 2B).

Potentiation of GSIS by anandamide was substantially diminished by the CB1R antagonist rimonabant at 10 μmol/L (Fig 2B), suggesting a CB1R-mediated process, at least in part. In fact, we confirmed mRNA expression of CB1R in intact pancreatic canine islets (Fig 2D). However, we found no differences in gene expression of CB1R, CB2R or TRPV1 between control-diet and HFD animals (CB1R: P = 1.00; CB2R: P = 0.57; TRPV1: P = 0.71).

### Physiological anandamide concentrations may not consistently stimulate insulin secretion ***in vitro***

**Fig 3:**
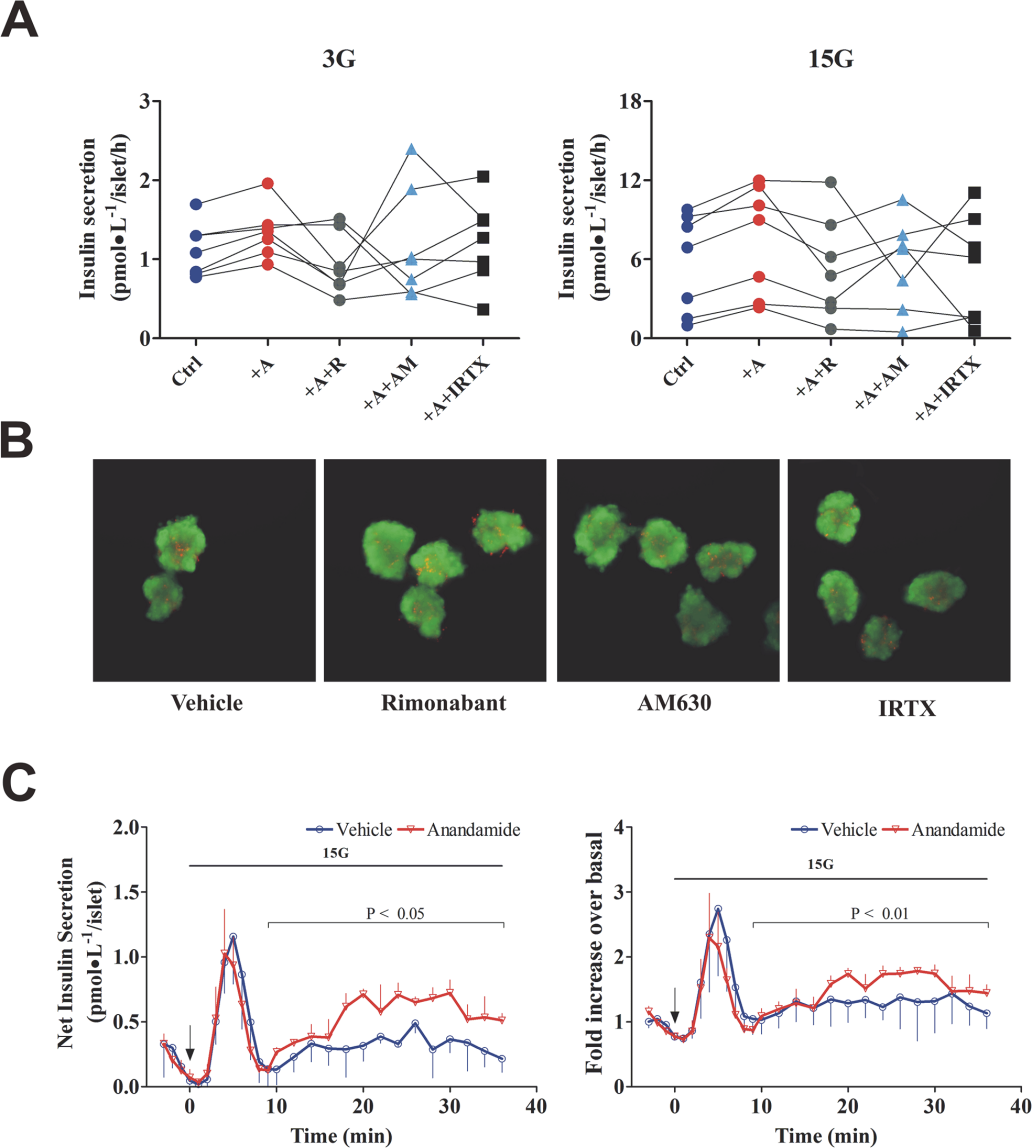
Physiological concentrations of anandamide enhance *in vitro* insulin secretion. (**A**) In islets from control-diet dogs (n = 7), anandamide significantly increased basal insulin secretion at 3 mmol/L glucose (3G) and GSIS at 15 mmol/L glucose (15G). Islets were incubated for 1 h with either anandamide (10 nmol/L) or cannabinoid receptor antagonists (100 nmol/L), as indicated. CB1R, CB2R, and TRPV1 antagonists rimonabant (R), AM630, and iodoresiniferatoxin (IRTX), respectively, were added prior to stimulation with high glucose and anandamide. Plots indicate the mean of 3–9 replicates for each dog. (**B**) When tested alone, none of the antagonist drugs had effect on islet viability compared with vehicle (n = 3). Total magnification: 100X. (**C**) Islets were continuously perifused with glucose 3 mmol/L and challenged with 15 mmol/L glucose (15G) from t = 0, as shown with the horizontal bar, in presence (n = 3) or absence (n = 3) of anandamide (10 nmol/L). Anandamide perifusion started concomitant with 15G, as indicated by the arrow. Plots of perifusion experiments represent means; bars represent S.E.M. P value represent the difference in overall profile between treatment and control during the second phase (t = 9–36 min). Analysis was performed using mixed-model linear regression to account for repeated measures.

In another set of control-diet animals (n = 7, body weight: 26.0±0.5 kg), we explored whether lower concentrations of anandamide, similar to those reported in plasma from moderate obese subjects [10], could stimulate insulin secretion in isolated islets. Islets incubated with 10 nmol/L anandamide did not show significant higher rates of basal insulin secretion (1.3±0.1 pmol•L^−1^/islet/h) as compared with no treatment (1.1±0.1 pmol•L^−1^/islet/h; P = 0.131, Friedman test) (Fig 3A). At same concentrations, anandamide-stimulated islets did not show significant higher GSIS (7.5±1.6 pmol•L^−1^/islet/h) as compared with non-stimulated islets (5.7±1.4 pmol•L^−1^/islet/h; P = 0.052, Friedman test). However, Fig 3A clearly shows a trend, in all dogs, for an increase in insulin secretion with anandamide, that appears to be decreased by rimonabant and not by the CB2R antagonist AM630 [26] or the TRPV1 antagonist iodoresiniferatoxin [27]. None of these cannabinoid antagonists impaired islet viability (Fig 3B).

Data from islet perifusion experiments showed an insulinotropic effect of low concentrations of anandamide. Although anandamide had no effect on the first phase (P = 0.198), it did increase the second phase of insulin secretion (P<0.01) (Fig 3C). These *in vitro* findings suggest that anandamide might play a physiological role in the regulation of insulin secretion.

### Anandamide-induced insulin secretion is dependent of paracrine regulation

**Fig 4:**
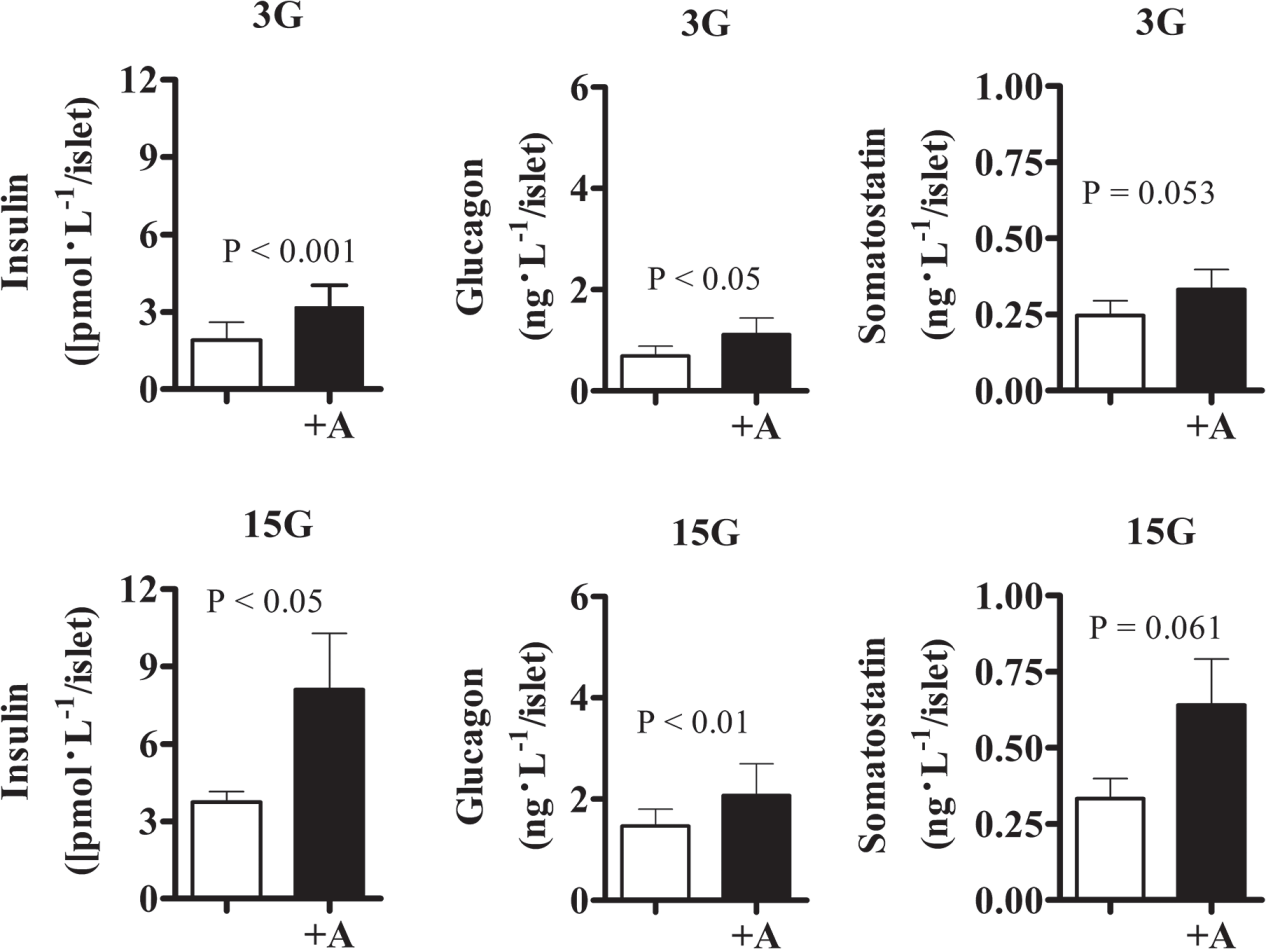
Anandamide significantly enhances insulin and glucagon secretion. Insulin, glucagon, and somatostatin concentrations were measured in batches of 100 islets from control-diet animals (n = 7) during static incubation with anandamide 10 μmol/L for 1 h. 3G, 3 mmol/L; 15G, 15 mmol/L glucose. Data are mean±S.E.M.

During static incubation, batches of 100 islets from control-diet animals (n = 7, body weight: 26.0±0.5 kg) incubated with 10 μmol/L anandamide for 1 h significantly increased basal insulin and glucagon secretion (Fig 4). Anandamide also potentiated GSIS and stimulated glucagon secretion. Similar results were found when batches of 15 islets from control-diet (n = 7, body weight: 28.8±1.0 kg) and HFD (n = 5, body weight: 34.2±1.6 kg) animals were incubated for 14 h with 10 μmol/L anandamide. However, we found no differences in anandamide-induced glucagon secretion between control and HFD animals at low glucose (5.5±2.1 and 3.6±0.9 ng•L^−1^/islet; P = 0.705) or high glucose concentrations (5.0±1.7 and 3.9±0.8 ng•L^−1^/islet; P = 0.705, control and HFD, respectively).

Using a regression model to determine the possible interaction among islet hormones, we found no association between anandamide and insulin secretion (P = 0.643) while controlling for glucose (P<0.001), glucagon (P<0.05), somatostatin (P<0.05), and type of diet (P = 0.059). Conversely, anandamide remained significantly associated with glucagon secretion (P<0.001) while controlling for glucose (P = 0.094), insulin (P = 0.054), somatostatin (P<0.05), and type of diet (P = 0.835). This analysis suggests that anandamide-stimulated insulin secretion is dependent on glucose concentration and paracrine effects, most likely glucagon since somatostatin did not significantly increase (Fig 4). Conversely, anandamide-stimulated glucagon secretion could result from a direct effect of this endocannabinoid on the pancreatic α-cells.

## Discussion

Although a direct association between fasting plasma anandamide levels and obesity has been demonstrated in humans [10, 11], whether this is a cause-effect relationship still remains unknown. In fact, no previous study has specifically studied the effect of high-fat diet-induced insulin resistance on plasma anandamide. Moreover, *in vitro* studies have demonstrated that anandamide stimulates insulin secretion in islets from lean rodents and humans [13, 14]. However, whether insulin resistance alters the insulinotropic effect of anandamide has remained unknown. In the present study, our findings in canines indicate that high-fat diet-induced insulin resistance does not increase plasma anandamide levels nor potentiate the insulinotropic effect of anandamide in isolated canine islets.

Human studies have shown elevated fasting plasma anandamide concentrations in moderate or severe obese subjects as compared with lean individuals [10, 11]. Conversely, insulin resistance appears to be associated with lower levels of anandamide, and increased levels of 2-AG and palmitoyl ethanolamide, at least in postmenopausal women [28]. In our canine model, despite a marked decrease in insulin sensitivity (by ~30%) and moderate body weight gain (by ~13%) after 22 weeks of HFD (Table 1), we found a significant decrease in fasting plasma 2-AG levels but not changes in the concentrations of anandamide or other endocannabinoids. Some explanations for these discrepancies include that the present study was interventional, whereas the previous studies were cross-sectional. Another explanation is the modest weight gain and absolute fat mass gain (~500 g) after fat feeding, although total abdominal fat depot increased by 75%. Although a previous study determined the effect of two weeks of fat feeding on human endocannabinoid levels, showing no changes in plasma anandamide or 2-AG levels [29], the diet used was isocaloric which resulted in no changes in body weight or fat mass. In addition, the latter study did not assess insulin sensitivity. It should be noted that elevation of anandamide levels in obesity does not seem to be consistent in all studies [30, 31], even despite severe obesity [31], suggesting that a cause-effect relationship might not exist, at least with BMI *per se*. Moreover, a rodent study exploring the effect of three weeks of fat feeding on anandamide levels showed elevated anandamide levels in the fat-fed animals [17]; however, the comparison between fat and chow diets was done between different groups but not within groups and assessment of insulin sensitivity was not performed. One interesting finding in the present study was the negative correlation of plasma 2-AG with plasma insulin and C-peptide, suggesting 2-AG could be associated with β-cell function rather than anandamide. However, further studies are required to elucidate any possible physiological role of 2-AG on β-cell function.

It does not appear to be clear whether insulin resistance *per se*, fat mass, or the type of fat diet is the major contributor to higher plasma endocannabinoid levels in obese individuals. Although the present study did not aim to investigate this, it should be noted that a diet rich in linoleic acid has been shown to increase endocannabinoid levels in the mouse brain [32]. In our study, we used a HFD that consisted of 53.0% fat, mainly from palmitic, oleic, and linoleic acids, in that order (http://nutritiondata.self.com). Interestingly, several other fat-rich diets may also increase anandamide and other endocannabinoid levels in the rat brain and liver [33]. Further studies are required to elucidate the main source of plasma endocannabinoid levels in obesity.

Although it can be argued that the absence of anandamide elevation is consistent with the lack of increase in β-cell function *in vivo*, supporting the concept that there is a link between plasma anandamide and insulin, this is unlikely, since a further analysis in a subset of dogs with fat-induced hyperinsulinemia showed no changes in plasma anandamide. These findings indicate that hyperinsulinemia does not require elevation of plasma anandamide. Since acute *in vivo* treatment with anandamide has been shown to decrease intraperitoneal glucose tolerance in wild type mice [34, 35] but not in CB1R^-/-^ mice [34], there is a possibility that elevation of plasma anandamide levels may be a cause rather than consequence (compensatory signal) of insulin resistance. Future studies exploring the chronic effect of anandamide treatment *in vivo* may help to elucidate this question.

The fact that the present study found no association between fasting plasma anandamide and insulin levels suggests that local anandamide concentrations, rather than circulating levels, could have a physiological role on islet hormone regulation, as anandamide can be synthesized in the exocrine and endocrine regions of the pancreas [14, 36, 37]. This is supported by previous studies [13, 14] and our findings in cultured canine islets, showing a consistent insulinotropic effect of anandamide in isolated pancreatic islets, both at supraphysiologic concentrations and in the nanomolar range, although to a lesser extent than those provoked by supraphysiologic concentrations. CB1R synthetic agonists have also been shown to stimulate insulin secretion in human islets [38]. Conversely, mouse studies have shown apparent contradicting results, where anandamide rather decreased insulin secretion [39, 40]. Although these divergent results could be species-related, it could also be explained by differences in the recovery time after islet isolation [41]. Anandamide seems to decrease insulin secretion in freshly isolated rat islets but enhance insulin secretion in rat islets cultured for 18–24 h prior to anandamide stimulation [41].

The fact that the CB1R antagonist rimonabant did not completely suppress the insulinotropic effect of anandamide in canine islets (Fig 2A and 2B) suggests that other receptors could be involved. In fact, anandamide is a non selective agonist of cannabinoid receptors [42–47], with a higher affinity and agonist effect for CB1R than for CB2R [48–51], and probably to a lesser extent an activator of other plasma membrane receptors including TRPV1 [52–54] and the G protein-coupled receptor 55 (GPR55) [55]. Anandamide can bind to CB2R at micromolar concentrations [46], whereas rimonabant can inhibit by ~40% the affinity of CB2R agonists at concentrations over 1 μmol/L [56], at least in Chinese hamster ovary cells. Thus, in our study, 10 μmol/L rimonabant could have blocked any possible effect of 10 μmol/L anandamide on CB2R. However, further experiments with anandamide at 10 nmol/L (Ki for CB2R = ≥280 nmol/L [3]), showed a trend for a decrease in anandamide effect by antagonism of CB1R and not by blocking CB2R or TRPV1 (Fig 3A). Nevertheless, our findings cannot prove the main receptor involved in the insulinotropic effect of anandamide.

Despite prolonged HFD resulting in a substantial increase in fat content and marked reduction in insulin sensitivity, islets from HFD animals did not exhibit further stimulation of basal insulin or GSIS by anandamide as compared with islets from control animals. These results, together with our findings *in vivo* showing that hyperinsulinemia, secondary to high-fat diet-induced insulin resistance, occurred in the absence of plasma anandamide elevation, do not support the notion that anandamide could contribute to hyperinsulinemia associated with insulin resistance or obesity. Of note, basal insulin in HFD islets was significantly lower compared with that in control islets (P = 0.010). Possible explanations for these findings are: 1) HFD islets incubated overnight prior to experiments *in vitro* could be exposed to higher insulin concentrations as compared with islets from control dogs, causing a downregulation of β-cell function, since insulin *per se* may exert a negative feedback on insulin secretion [57]; 2) HFD islets could have similar rates of insulin secretion than control islets, if secretion is normalized to islet size. Indeed, previous studies in Zucker rat [58] and humans islets [59] have shown similar basal insulin and glucose-stimulated insulin secretion (GSIS) between islets from lean and fat animals when normalized to islet diameter or cell number. The first possibility could explain the lower basal insulin in the HFD group, and the second possibility could explain the lack of differences in GSIS in our study. However, this remains speculative.

One intriguing finding was that anandamide enhanced GSIS by stimulating the second phase of insulin release. This is consistent with the potentiation of the second phase of insulin release by fatty acids reported in mouse and rat islets [60, 61]. We also found a stimulatory effect of anandamide on glucagon secretion, suggesting that the endocannabinoid anandamide may play a physiological role in islet hormone regulation. Increased glucagon secretion has also been shown in response to the specific CB1R synthetic agonist arachidonoyl-2′-chloroethylamide in human [14] and mouse islets [62]. The fact that we found no differences between HFD and control animals in the stimulatory effect of anandamide on insulin and glucagon, is supported by the similar mRNA expression of CB1R and CB2R between these groups. Although mRNA expression of cannabinoid receptors has been demonstrated in other species [14, 40, 62–69], no study has compared their expression between lean and obese or insulin resistant animals.

We used a regression model to elucidate the interaction among islet hormones in response to anandamide *in vitro*. This analysis suggests that anandamide stimulation of insulin secretion is dependent on paracrine regulation, most likely glucagon. These findings are consistent with the insulinotropic effect of glucagon in rodent islets [70] and dog pancreas *ex vivo* [71]. This approach also showed that anandamide stimulates glucagon secretion independent of paracrine regulation, regardless of glucose concentration, suggesting a possible direct effect of anandamide on α-cells. These data need to be confirmed in future studies using pure preparations of α-cells and specific antibodies for islet hormone receptors.

Our study has some limitations. The present study was performed in a small number of dogs. Although we used a well established canine model of high-fat diet induced insulin resistance, 22 weeks of fat feeding in canines resulted in a mild increase in body weight and absolute fat mass, which was limited to the abdominal region. Whether a more pronounced weight gain and fat mass accumulation may induce significant changes in anandamide levels remains unknown. In addition, we cannot exclude the possibility that whole-body fat mass could be correlated with plasma anandamide or other endocannabinoids in our study. Although we measured several biochemical parameters in plasma, including most known endocannabinoids, these were measured in fasting conditions only. It is possible that elevation of anandamide levels under post-prandial conditions or during nighttime may have occurred. For example, plasma anandamide levels have shown to be higher at night as compared with daytime in mice [17]. Another limitation is that we were not able to test the changes in β-cell function *in vitro* in the same animals. Finally, since the effects of anandamide were tested *in vitro*, but not after anandamide administration *in vivo*, we cannot completely prove a physiological role of anandamide on insulin secretion.

In conclusion, our results clearly demonstrate that anandamide stimulates insulin and glucagon secretion in cultured pancreatic canine islets from both HFD- and control-diet animals. However, high-fat diet-induced insulin resistance did not alter plasma anandamide levels or further potentiate the insulinotropic effect of anandamide *in vitro*.
