# Corrections: High-Fat Diet-Induced Insulin Resistance does not Increase Plasma Anandamide Levels or Potentiate Anandamide Insulinotropic Effect in Isolated Canine Islets

**DOI:** 10.1371/journal.pone.0131033

**Published:** 2015-06-15

**Authors:** Orison O. Woolcott, Joyce M. Richey, Morvarid Kabir, Robert H. Chow, Malini S. Iyer, Erlinda L. Kirkman, Darko Stefanovski, Maya Lottati, Stella P. Kim, L. Nicole Harrison, Viorica Ionut, Dan Zheng, Isabel R. Hsu, Karyn J. Catalano, Jenny D. Chiu, Heather Bradshaw, Qiang Wu, Cathryn M. Kolka, Richard N. Bergman

Cathryn M. Kolka is not included in the author byline. She should be listed as the eighteenth author and affiliated with Diabetes and Obesity Research Institute, Cedars-Sinai Medical Center, Los Angeles, California, USA.
